# Monophosphate Derivatives of Luteolin and Apigenin as Efficient Precursors with Improved Oral Bioavailability in Rats

**DOI:** 10.3390/antiox13121530

**Published:** 2024-12-13

**Authors:** Sydney Wu, Shang-Ta Wang, Guan-Yuan Chen, Chen Hsu, Yi-Hsin Chen, Hsin-Ya Tsai, Te-I Weng, Chien-Li Chen, Yi-Fang Wu, Nan-Wei Su

**Affiliations:** 1 Department of Agricultural Chemistry, National Taiwan University, Taipei 106, Taiwan; r10623034@ntu.edu.tw (S.W.); d04623002@ntu.edu.tw (C.H.); d08623003@ntu.edu.tw (H.-Y.T.); 2 Department of Food Science, National Taiwan Ocean University, Keelung 202, Taiwan; wst@mail.ntou.edu.tw (S.-T.W.); sander.chen@mail.ntou.edu.tw (C.-L.C.); 3 Institute of Food Safety and Risk Management, National Taiwan Ocean University, Keelung 202, Taiwan; 4 Forensic and Clinical Toxicology Center, National Taiwan University Hospital, Taipei 100, Taiwan; gychen@ntu.edu.tw (G.-Y.C.); wengtei2@ntu.edu.tw (T.-I.W.); lorlan11501@gmail.com (Y.-F.W.); 5 Department and Graduate Institute of Forensic Medicine, College of Medicine, National Taiwan University, Taipei 100, Taiwan; 6 Department of Biochemical Science & Technology, National Taiwan University, Taipei 106, Taiwan; r09b22038@ntu.edu.tw; 7 Department of Emergency Medicine, National Taiwan University Hospital, Taipei 100, Taiwan; 8 School of Pharmacy, College of Medicine, National Taiwan University, Taipei 100, Taiwan

**Keywords:** bioconversion, flavone, *Bacillus subtilis*, bioavailability, phosphorylation, phosphate prodrug

## Abstract

Luteolin (Lut) and apigenin (Apn), flavones present in various edible plants, exhibit diverse antioxidant and pharmacological activities but have limited in vivo efficacy due to low water solubility and poor bioavailability. Here, we generated luteolin and apigenin monophosphate derivatives (LutPs and ApnPs) individually via microbial biotransformation. We then characterized their physicochemical properties and evaluated their in vitro and in vivo pharmacokinetics and bioavailability. Both LutPs and ApnPs showed enhanced solubility and dissolution and remained stable in simulated gastrointestinal conditions. Additionally, they efficiently reverted to parental forms via alkaline phosphatase in Caco-2 cells. Following oral administration in rats, LutPs and ApnPs exhibited higher plasma exposure to both aglycone and conjugated forms compared to Lut and Apn. Notably, the in vivo biotransformation of Apn to Lut was observed in all apigenin-related groups. Our study suggests that flavone monophosphates are effective alternatives with enhanced bioavailability, providing insights for the potential application of emerging bioactive nutraceuticals.

## 1. Introduction

Flavones, a subclass of flavonoids, are widely found in edible herbs such as chamomile, celery, parsley, and sweet peppers [1]. Among these plants, luteolin (3′,4′,5,7-tetrahydroxyflavone, Lut) and apigenin (4′,5,7-trihydroxyflavone, Apn) are two of the most prevalent flavones and exhibit a wide range of pharmacological activities. They have garnered significant interest for their foundational antioxidant and anti-inflammatory activities at the molecular level, which may be attributed to the presence of multiple hydroxy groups of flavone skeleton. These structural features enable them to neutralize reactive oxygen species (ROS) and modulate the cellular redox signaling pathway [2,3,4], which play a critical role in preventing oxidative damage and inflammation [5]. Lut has been shown to cross the blood–brain barrier, providing significant neuroprotective effects by eliminating oxidative stress and mitigating apoptosis. Studies have shown that the intraperitoneal injection of Lut in rats at doses of 10 and 25 mg/kg effectively protects the brain from ischemic damage by upregulating antioxidant enzymes, including superoxide dismutase (SOD) and glutathione peroxidase (GPx) [6,7]. Additionally, Lut has been identified as a senomorphic compound capable of disrupting p16–CDK6 interaction, a key mechanism involved in aging and age-related diseases [8]. Similarly, Apn exhibits strong antioxidant activity by scavenging free radicals and enhancing antioxidant defense mechanisms through multiple pathways. Apn has been reported to activate the nuclear factor erythroid 2-related factor 2 (Nrf2) pathway, thereby increasing the expression of downstream proteins such as heme oxygenase 1 (HO-1) and NAD(P)H quinone oxidoreductase 1 (NQO1), which prevents oxidative stress-induced DNA damage and metabolic syndrome [9,10]. Moreover, Apn has shown efficacy in inhibiting tumor cell proliferation and metastasis in breast [11] and prostate cancers [12]. These studies suggest that Lut and Apn hold therapeutic potential in managing oxidative stress-related diseases, including neurodegenerative disorders, cardiovascular diseases, and cancers, attributed to their antioxidant properties.

However, despite their promising antioxidant capacity, concerns have been raised about the bioavailability of Lut and Apn. In practice, the clinical application of these compounds is severely limited by their low bioavailability, which may impair their antioxidation efficacy and hinder a comprehensive understanding of their biological behavior [13]. Pharmacokinetic studies have confirmed that Lut and Apn exhibit poor absolute oral bioavailability, with values of only 4.10% and 0.71%, respectively [14,15]. In the case of Lut, the administration of 20 μmol/kg (equivalent to 5.725 mg/kg) via gavage in rats resulted in brain tissue concentrations of less than 0.1 nmol/g [16], a level far below the threshold required to exert antioxidant effects on neural cells, which is commonly recognized to be around 20 μM. Previous research demonstrated that the treatment of PC12 cells with 20 μM of Lut stimulated neurite outgrowth by promoting the binding of nuclear factor E2-related factor 2 (Nrf2) to the antioxidant response element (ARE), an enhancer sequence in the heme oxygenase-1 (HO-1) promoter, providing a clear example of its potential antioxidation and neuroprotective tissue level [17]. Apn faces similar challenges. Research indicates that the oral administration of 10 mg/kg Apn in rats yielded a maximum plasma concentration (C_max_) of only 0.08 μM [18], making it difficult to achieve effective therapeutic levels. In embryonic mouse heart cells treated with Apn at concentrations of around 10 μM, it effectively regulated inflammatory responses in a dose-dependent manner by reducing cyclooxygenase (COX)-2 expression, a key enzyme involved in various inflammatory diseases [19], providing another example of its effective systemic level. Therefore, developing strategies to enhance the bioavailability of these flavones is crucial to achieving the systemic and/or tissue concentrations required for their antioxidant and therapeutic efficacy.

To address the aforementioned issue, many studies have been committed to develop formulation technologies such as nano-crystallization [20,21], liposome encapsulation [22,23], polymer micelles [24,25], and self-nanoemulsifying drug delivery systems [26,27]. Despite these technological advancements, the successful translation of these methods into practical clinical applications has been limited.

In our previous research, we discovered a distinct bioconversion mediated by *Bacillus subtilis* BCRC 80517, which converts genistein, hesperetin, and Lut into the corresponding monophosphate derivatives through co-culturing. These phosphate derivatives align with the phosphate prodrug strategy widely used in the pharmaceutical industry. By conjugating a phosphate group to the parent compound, this approach addresses solubility challenges and further enhances oral bioavailability. The conversion of the derivatives into their parent form by epithelial membrane-associated alkaline phosphatase (ALP) increases the local concentration near the mucosal membrane. This conversion can lead to local supersaturation, generating a stronger concentration gradient and improving the absorptive flux of the parent form. The enhanced solubility may also saturate intestinal efflux transporters and metabolic pathways, further promoting transepithelial flux and increasing systemic exposure [7,28,29,30]. Additionally, the oral administration of genistein monophosphate has demonstrated significant efficacy in preventing the loss of bone mineral density and reducing Young’s modulus in ovariectomized rats, as well as improving bone architecture [31].

In this study, we generated three luteolin phosphate derivatives (LutPs) and two apigenin phosphate derivatives (ApnPs) by co-culturing *Bacillus substilis* BCRC 80517 with the native aglyconic flavones for biophosphorylation. We conducted pharmacokinetic studies to evaluate their metabolic fate in rats. Experimental groups included the native Lut and Apn, as well as their phosphorylated products—LutPs and ApnPs—along with naturally occurring forms such as luteolin 7-*O*-glucoside (Lut7G) and apigenin 7-*O*-glucoside (Apn7G). Our aim was to investigate whether these flavone monophosphate derivatives could be promising alternatives for enhancing oral bioavailability, providing a potential strategy for their application as effective antioxidants.

## 2. Materials and Methods

### 2.1. Materials

Luteolin (3′,4′,5,7-tetrahydroxyflavone, HPLC ≥ 98%) was obtained from Shaanxi Creative Herb Biotechnology Co., Ltd. (Xi’an, China), and apigenin (4′,5,7-trihydroxyflavone, HPLC ≥ 98%) was obtained from Huike Botanical Development Co., Ltd. (Xi’an, China). Luteolin 7-*O*-glucoside and apigenin 7-*O*-glucoside (both HPLC ≥ 98%) were purchased from ChemFaces (Wuhan, China). Fosphenytoin sodium, *β*-glucuronidase (Helix pomatia, Type H-5), pancreatin (from porcine pancreas, 350 FIP-U/g protease, 6000 FIP-U/g lipase, 7500 FIP-U/g amylase,), and the minimum essential medium (MEM) were from Sigma Aldrich (St. Louis, MO, USA). The nutrient broth (NB) medium was from Difco Laboratories (Detroit, MI, USA). The antibiotic antimycotic solution (10,000 I.U./mL penicillin, 10 mg/mL streptomycin, 25 μg/mL amphotericin), trypsin-EDTA (0.25%), and fetal bovine serum (FBS) were from Gibco-BRL (New York, NY, USA). Heparin sodium salt was from TCI (Tokyo, Japan).

### 2.2. Preparation of LutPs and ApnPs

LutPs and ApnPs were produced through a bioconversion process that involved culturing *Bacillus subtilis* BCRC 80517 with Lut and Apn, respectively. Briefly, a 500 mL Hinton’s flask containing 100 mL of fresh nutrition broth (NB) was inoculated with 5% primary seed culture and incubated at 37 °C, 150 rpm, for 12 h as the secondary seed culture. Each 500 mL flask contained 85 mL of fresh NB and 10 mL of tested flavone suspension at 20 mg/mL, and 5 mL of inoculum from the secondary seed culture was incubated at 37 °C at 150 rpm for 48 h. LutPs and ApnPs were recovered firstly by extraction with a 2-fold volume of ethyl acetate three times from the acidified harvest culture broth. The ethyl acetate extracts were collected for further purification. Then, the following procedures were conducted as described by Wang et al. [29]. Briefly, an Isco CombiFlash Rf150 Purification System (Teledyne Isco, Lincoln, NE, USA) equipped with a prepacked C-18 column was used to separately collect the fractions containing LutPs-dominant eluate and ApnPs-dominant eluate. After extensive washing to remove impurities, the column was eluted sequentially with a solution containing increasing levels of methanol, and the active fractions of LutPs-dominant with strong absorption at 354 nm and for ApnPs-dominant with absorption at 270 nm were collected, respectively. After evaporation to remove methanol under reduced pressure, the isolated LutPs and ApnPs were re-dissolved in water and subjected to lyophilization. The lyophilized powders were prepared for further uses. The assessment of aqueous solubility was conducted via the classical shake-flask method, meaning that a certain amount of excess samples was added to ultrapure water (≥18 MΩ·cm) and incubated at constant 25 °C overnight with occasional shaking to reach equilibrium. Then, the resulting supernatant (the saturated solution) was aliquoted and diluted carefully and properly to perform the quantitative analysis using HPLC. Additionally, ChemDraw Professional 16.0 (PerkinElmer, Norwalk, CT, USA) was used to predict the cLogP values of the compounds based on their chemical structures.

### 2.3. Stability Assay in Simulated Gastric Fluid (SGF) and Simulated Intestinal Fluid (SIF)

For the SGF solution, 5 g of pepsin was dissolved in 1 L of deionized water with the addition of hydrochloric acid to achieve a pH of 1.8. The SIF solution consisted of 6.8 g of monopotassium phosphate and 10 g of pancreatin dissolved in 1 L of deionized water, maintaining a pH of 6.8. To assess the stability of LutPs, Lut, ApnPs, and Apn, each at a concentration of 125 mg/L in SGF and SIF, samples were incubated at 37 °C for 240 min with gentle stirring at 150 rpm. Samples were collected at specific time intervals (0, 15, 30, 60, 90, 120, 180, and 240 min), immediately mixed with an equal volume of methanol, and centrifuged at 4 °C under 21,500× *g* for 10 min. The resulting supernatants were then filtered through a 0.22 μm filter and subjected to analysis using HPLC to determine the remaining content of LutPs, Lut, ApnPs, and Apn.

### 2.4. Dissolution Study

The dissolution properties of Lut, Apn, and their phosphate derivatives were evaluated using a USP 32 apparatus II (paddle method) in an AT Xtend dissolution tester (SOTAX Co., Westborough, MA, USA), in accordance with the US Pharmacopoeia XXII general method. Due to the different molecular masses of Lut, Apn, and their derivatives, their dissolution profiles were assessed at a concentration of 20 μM in 900 mL of 0.05 M phosphate buffer, at a pH of 6.8 and a temperature of 37 °C under a rotational speed of 50 rpm. To investigate the influence of bile extract (comprising bile salts and phospholipids) on the dissolution of these compounds, bile extract solutions were prepared according to the protocol described by Makioka et al. [32] This involved dissolving 12 g of porcine bile extract and 8.4 g of sodium bicarbonate in 1 L of deionized water, which served as the dissolution medium. After the sample was placed in the dissolution medium, 1 mL aliquots of the medium were collected at 5, 10, 20, 30, 45, and 60 min of incubation, and filtered through a 0.2 μm polyethersulfone filter (Pall Corp., New York, NY, USA). Subsequently, 500 μL of each sample was acidified with 5 μL of 2 M acetic acid and stored at −20 °C until HPLC analysis.

### 2.5. Dephosphorylation Assay by Caco-2 Cell Apical Membrane-Associated ALP

The Caco-2 cells (human colon adenocarcinoma, clone of Caco-2) were obtained from the Bioresource Collection and Research Center, Hsinchu, Taiwan. Caco-2 cells were cultured according to previously established protocols [29]. For the dephosphorylation study, the cells were seeded onto 6-well plates at a density of 3.0 × 10^5^ cells per well in 2 mL of fresh medium. The medium was refreshed every 48 h. After 5 days of incubation, the medium was substituted with 2 mL of Hank’s Balanced Salt Solution (HBSS) containing 20 μM of LutPs or 50 μM of ApnPs. The plates were then incubated at 37 °C, and aliquots of 200 μL were collected at 15, 30, 60, and 120 min. Each aliquot was mixed with 200 μL of methanol, followed by centrifugation at 21,500× *g* for 10 min at 4 °C. The supernatants were analyzed by HPLC to quantify the relative content of LutPs to Lut and ApnPs to Apn. To assess the activity of human intestinal alkaline phosphatase (ALP), a positive control of fosphenytoin, a phenytoin phosphate prodrug, was used. The elimination half-life (T_1/2_) of dephosphorylation was determined using Equation (1):

T_1/2_ = −0.693/k (1)

where 0.693 is approximately the natural logarithm of 2, and k is the slope of the linear regression of the dephosphorylation curve.

### 2.6. Quantification by HPLC

The HPLC system employed in this study consisted of the Waters Alliance 2695 separation module (Waters, Milford, MA, USA), a YMC-Pack ODS-AM C_18_ column (4.6 × 250 mm, 5 µm) with a guard cartridge (Hichrom 5C18, Berkshire, UK) and a Waters 2487 Dual-Absorbance Detector (Waters, Milford, MA, USA). Chromatographic separation was conducted at a temperature of 30 °C, utilizing a linear gradient mobile phase consisting of 0.1% phosphoric acid in H_2_O (solvent A) and 0.1% phosphoric acid in acetonitrile (solvent B). The elution gradient programs for quantifying each compound are detailed in Supplementary Materials Table S1. Luteolin and its derivatives were detected at a wavelength of 354 nm, while apigenin and its derivatives were detected at a wavelength of 270 nm, and fosphenytoin at 210 nm. The quantitation was conducted using the external standard method with calibration curves, and the quality was ensured by maintaining an R^2^ value exceeding 0.99.

### 2.7. Oral Bioavailability in Sprague Dawley (SD) Rats

The protocols were approved by the Institutional Animal Care and Use Committees of National Taiwan University (NTU, project no. NTU-107-EL-00222) for the tests of Lut, Lut7G, and LutPs and the National Taiwan Ocean University (NTOU, project no. 111026) for the tests of Apn, Apn7G, and ApnPs. Male SD rats (250 ± 10 g, eight weeks old) were obtained from BioLASCO Taiwan Co. (Taipei, Taiwan). and housed individually. The facilities were maintained at 25 °C, at constant humidity, and a 12 h light/dark cycle with ad libitum access to food and water and acclimated for at least 7 days prior to any experiments. Rats were fasted for 24 h before the experiments, with water freely available. For the pharmacokinetic study, rats were randomly assigned to three groups for the tests (*n* = 4 for Lut-relevant groups; *n* = 4–5 for Apn-relevant groups). The Lut-relevant groups received Lut aqueous suspension, Lut7G aqueous suspension, or LutPs solution. The Apn-relevant groups received Apn aqueous suspension, Apn7G aqueous suspension, or ApnPs solution. Dosages were designed as 174.7 µmol/kg for Lut and 185.02 µmol/kg for Apn, based on the human dietary supplement dosage of 500 mg/day, translated by the body surface area index as per FDA guidelines [33]. Blood samples (300 µL) were collected from the jugular vein at various times using heparinized tubes, and plasma was obtained by centrifugation (4 °C, 3000× *g*, 10 min). Lut and Apn were quantified in biological samples after enzymatic hydrolysis of their conjugated forms. The plasma was acidified to pH 5 by adding 10 μL of 0.5 M acetate buffer. The preheated reaction mixture of 10 μL of β-glucuronidase (with glucuronidase, sulfatase, and glucosidase activities, 200 units) and 100 μL of plasma (spiked with genistein at 20 μg/mL as the internal standard) was then incubated at 37 °C for 1 h. The mixture was fractionated using a Sep-Pak C18 cartridge (Waters, Milford, MA, USA), eluted in order with 0.01 M oxalic acid, water/methanol/0.5 M oxalic acid (88:10:2), distilled water, and 100% methanol. The methanol fraction was adjusted to a constant volume of 500 μL and filtered with a 0.22 μm membrane to remove insoluble matter, and, subsequently, the filtrate underwent UPLC-MS/MS.

The levels of Lut and Apn in the plasma were quantified using a Waters ACQUITY UPLC I-Class system (Waters, Milford, MA, USA) equipped with an XEVO TQS-microTriple-Quadrupole Tandem Mass Spectrometer (Waters, Milford, MA, USA). Chromatographic separation was achieved using a Thermo Hypersil GOLD aQ C18 (50 × 2.1 mm, 1.9 μm) column (Thermo Fisher Scientific, Waltham, MA, USA). The injection volume was 3 μL. After samples were introduced, solvent B was held isocratic at 50% over 1 min, increased to 99% over 1.2 min, held isocratic at 99% over 2.8 min, decreased to 50% over 3 min, and finally held isocratic at 50% for 1 min, at a flow rate of 0.4 mL/min. The temperature of the column oven was set at 40 °C. The MRM mode in negative mode was used, and the MS parameter was set as follows. The most intense precursor-to-product transitions were selected: *m*/*z* 285→133 for luteolin, and *m*/*z* 269→107 and *m*/*z* 269→41 for apigenin and genistein, respectively. The source voltage was 1500 V. The desolvation temperature was 400 °C, and the desolvation gas flow was 800 L/h. The source temperature was 150 °C. The contents of Lut and Apn were calculated by the calibration curve and the response obtained by the peak area of them against the internal standard. Details on the multiple reaction monitoring (MRM) conditions, elution gradient programs, and UPLC method validation are provided in the Supplementary Materials Tables S2 and S3. The pharmacokinetic parameters, including maximum concentration (C_max_), time to reach maximum concentration (T_max_), and area under curve (AUC), were determined using the pharmacokinetic software WinNonlin Standard Edition v1.1 (Pharsight Corp., Mountainview, CA, USA).

### 2.8. Statistical Analysis

All statistical analyses of the samples were conducted with GraphPad Prism 9 (GraphPad Software, San Diego, CA, USA). Significant differences were assessed with a one-way ANOVA followed by Tukey’s multiple comparison test (*p* < 0.05).

## 3. Results

### 3.1. Physicochemical Properties of LutPs and ApnPs

**Figure 1:**
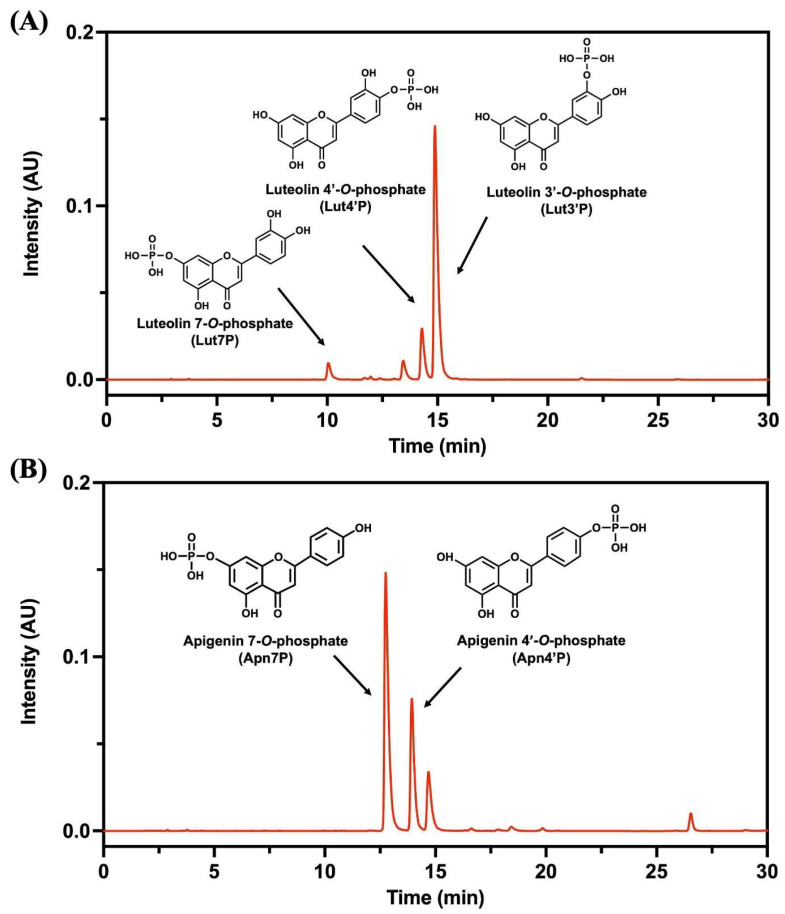
HPLC chromatograms of (**A**) LutPs, with Lut3′P at a content of 665.0 mg/g, Lut4′P at 191.3 mg/g, and Lut7P at 54.7 mg/g, and (**B**) ApnPs, with Apn7P at 642.2 mg/g and Apn4′P at 272.9 mg/g, obtained through the flash chromatography process. Lut4′P, luteolin 4′-*O*-phosphate; Lut3′P, luteolin 3′-*O*-phosphate; Apn4′P, apigenin 4′-*O*-phosphate; and Apn7′P, apigenin 7′-*O*-phosphate.

The purified test samples of LutPs and ApnPs from microbial phosphorylation were analyzed for their components using our previously established HPLC method, outlined by Hsu et al. [34] Details on the quantitative analysis and individual content were shown in Supplementary Materials Table S4. Figure 1 presents the chromatograms of the obtained LutPs and ApnPs. The LutPs consisted of luteolin 7-*O*-phosphate (Lut7P), luteolin 4′-*O*-phosphate (Lut4′P), and luteolin 3′-*O*-phosphate (Lut3′P), and Lut3′P was the predominant constituent at 665.0 mg/g, while Lut4′P and Lut7P were present at 191.3 mg/g and 54.7 mg/g, respectively. The ApnPs, consisting of apigenin 7-*O*-phosphate (Apn7P) and apigenin 4′-*O*-phosphate (Apn4′P), were quantified at 642.2 mg/g and 272.9 mg/g, respectively.

We further evaluated the octanol–water partition coefficients (clogPs) of LutPs and ApnPs using ChemDraw Professional 16.0 (PerkinElmer, Norwalk, CT, USA). The clogP is a critical indicator commonly used to assess the aqueous solubility of organic compounds. The clogP values for aglyconic Lut and Apn were 1.78 and 2.38, respectively. In contrast, their phosphate derivatives had the following values: Lut7P (0.44), Lut3′P (0.37), Lut4′P (0.37), Apn7P (1.04), and Apn4′P (1.04). These alterations represented substantial improvements in their aqueous solubility, with LutPs and ApnPs achieving solubility increases of approximately 1380-fold and 15,000-fold, respectively, compared to their aglycone forms (Table 1).

### 3.2. In Vitro Digestibility and Dissolution Profiles of LutPs and ApnPs

**Figure 2:**
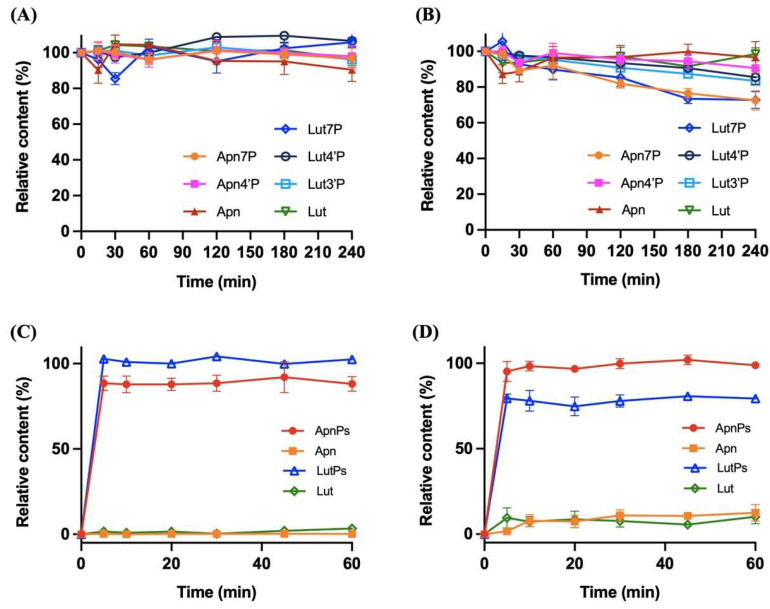
The physiological digestive stability of Lut, LutPs, Apn, and ApnPs at a concentration of 125 mg/L in (**A**) simulated gastric fluid and (**B**) simulated intestinal fluid, with an incubation period of 240 min, and dissolution profiles of Lut, LutPs, Apn, and ApnPs in (**C**) a pH of 6.8 in phosphate buffer alone and in (**D**) bile salt solution. Data are the mean ± SD (*n* = 3). Lut, luteolin; Lut4′P, luteolin 4′-*O*-phosphate; Lut3′P, luteolin 3′-*O*-phosphate; Lut7P, luteolin 7-*O*-phosphate; Apn, apigenin; Apn4′P, apigenin 4′-*O*-phosphate; and Apn7′P, apigenin 7′-*O*-phosphate.

To further investigate the stability of the compounds under gastric and intestinal physiological conditions, we utilized simulated gastric fluid (SGF) with pepsin at pH 1.5 and simulated intestinal fluid (SIF) with pancreatin at pH 6.8, representing enzymatic hydrolysis in the stomach and intestines. As shown in Figure 2A,B, we demonstrated that Lut3′P, Lut4′P, and Apn4′P retained over 80% of their relative content after 240 min of incubation, while Lut7P and Apn7P retained 73% and 76% of their content, respectively.

The dissolution tests further highlighted the enhanced performance of the phosphate derivatives. In standard dissolution media, LutPs and ApnPs achieved complete dissolution within 5 min, significantly surpassing the native compounds where Lut and Apn only reached dissolution rates of 3.5% and 0.24%, respectively, after 60 min of incubation (Figure 2C). Meanwhile, the bile extract solution improved the dissolution rates, with Apn and ApnPs increasing to 12.5% and 98.8%, respectively. For Lut, the rate improved to 10.2%, but the addition of bile extract had an adverse effect on LutPs (Figure 2D). It is noteworthy that Lut may exhibit a high binding capacity toward amino acids and bile salts, which could potentially lead to a loss during the sample preparation process, thereby reducing the dissolution rate of LutPs.

**Figure 3:**
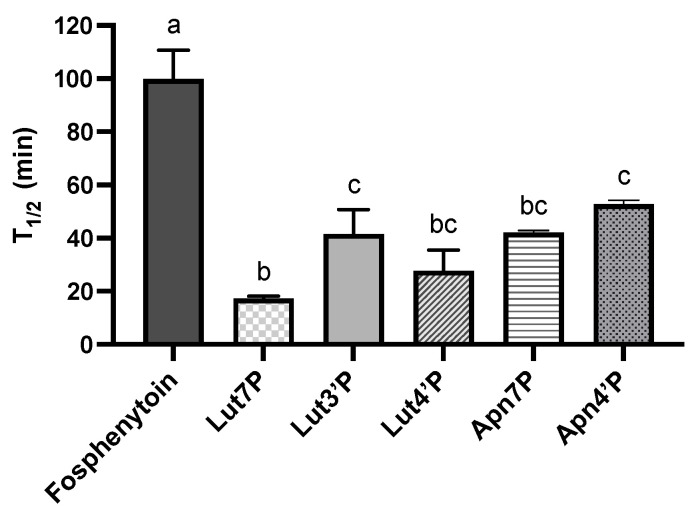
The elimination half-life (T_1/2_) of tested compounds at an initial concentration of 20 μM by apical membrane-associated alkaline phosphatase with 120 min of incubation. Data are the mean ± SD (*n* = 3). The elimination half-life was calculated as T_1/2_ = −0.693/k. Values with different letters are significantly different by one-way ANOVA followed by Tukey’s multiple comparison test (*p* < 0.05). LutPs, luteolin phosphate derivatives; Lut4′P, luteolin 4′-*O*-phosphate; Lut3′P, luteolin 3′-*O*-phosphate; Lut7P, luteolin 7-*O*-phosphate; Apn4′P, apigenin 4′-*O*-phosphate; and Apn7′P, apigenin 7′-*O*-phosphate.

Furthermore, we used a Caco-2 cell monolayer model to investigate the dephosphorylation efficiency of LutPs and ApnPs by human ALP. To evaluate this efficiency, fosphenytoin—a phosphate prodrug commonly prescribed for the treatment of convulsive status epilepticus—served as a positive reference due to its rapid dephosphorylation by intestinal ALP. By calculating the elimination half-life (T_1/2_), we found that Lut7P, Lut3′P, Lut4′P, Apn7P, Apn4′P, and fosphenytoin displayed T_1/2_ values of 17.2, 41.6, 27.8, 43.8, 64.1, and 99.9 min, respectively (Figure 3). The dephosphorylation efficiency of LutPs and ApnPs exceeded that of fosphenytoin, suggesting that LutPs and ApnPs exhibit high affinity for human ALP.

### 3.3. Pharmacokinetic Studies of Lut and Its Derivatives

Pharmacokinetic studies were conducted to evaluate the oral absorption of LutPs by quantifying the plasma concentrations of Lut and its phase II metabolites. The absorption profiles of LutPs were compared to those of Lut7G and native Lut.

**Figure 4:**
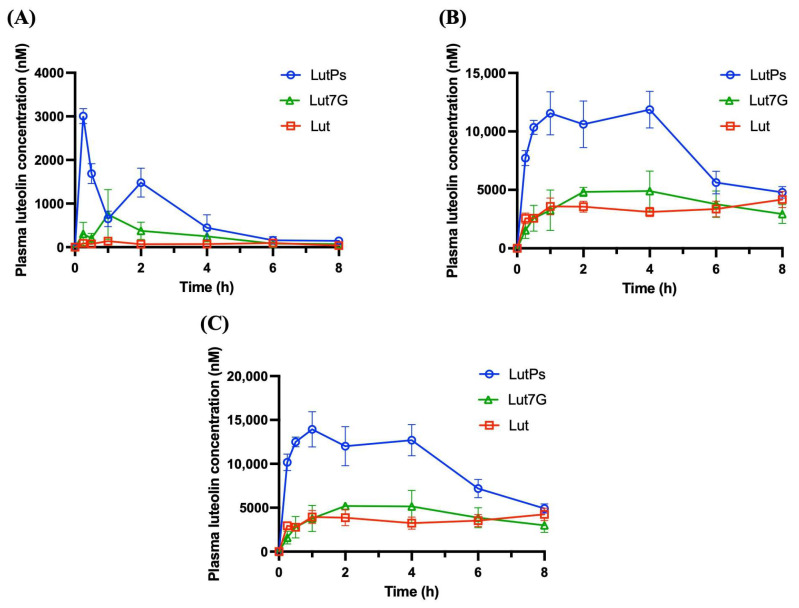
Mean plasma concentration–time profiles of (**A**) aglyconic Lut, (**B**) Lut conjugates, and (**C**) sum of Lut and its conjugates in rats after the oral administration of Lut, Lut7G suspension, and LutPs solution at 174.67 μmol/kg B.W. Data are the mean ± SE (*n* = 3). Lut, luteolin; LutPs, luteolin phosphate derivatives; and Lut7G, luteolin 7-*O*-glucoside.

In Figure 4A, the pharmacokinetic profiles reveal that aglyconic Lut exhibited the lowest absorption rate, whereas LutPs showed the highest. Table 2 indicates that the LutPs group exhibited rapid absorption with the shortest time to reach maximum concentration (T_max_) value of 0.25 h, while both the Lut7G and the Lut group displayed delayed absorption, each with a T_max_ value of 1 h. Moreover, the LutPs group exhibited the highest plasma levels, demonstrating almost 8.6- and 2.7-fold higher values in the area under the plasma concentration–time curve (AUC_0–t_) and 21.5- and 4.0-fold higher maximum concentration (C_max_) value compared to the Lut and Lut7G group, respectively. Notably, the absorption fraction of aglyconic Lut in the LutPs group showed a double-peak phenomenon at 0.25 and 2 h, whereas the Lut group exhibited peaks at 1 and 6 h. This pattern is consistent with the findings of previous studies, suggesting active and efficient entero-hepatic (or enterocytic) recycling of Lut [35]. Figure 4B shows the plasma levels of phase II metabolites of Lut. The concentrations of conjugated metabolites were substantially higher than those of aglyconic Lut in the plasma, particularly in the Lut group. It is noteworthy that, when considering phase II metabolites, the C_max_ of the LutPs group was delayed to 60 min, indicating that the hydrolysis of LutPs, facilitated by enterocyte ALP, allowed for the rapid accumulation of Lut in the cell membrane. This facilitated the entry of numerous untransformed Lut into the systemic circulation without being affected by first-pass metabolism. Figure 4C presents the overall plasma concentrations of Lut for each group. The AUC_0–∞_ values for LutPs, Lut, and Lut7G were 83859, 31659, and 35419 nM·h, respectively (Table 2). Notably, LutPs exhibited 2.65- and 2.36-fold higher bioavailability than the Lut and Lut7G groups, respectively.

### 3.4. Pharmacokinetic Studies of Apn and Its Derivatives

**Figure 5:**
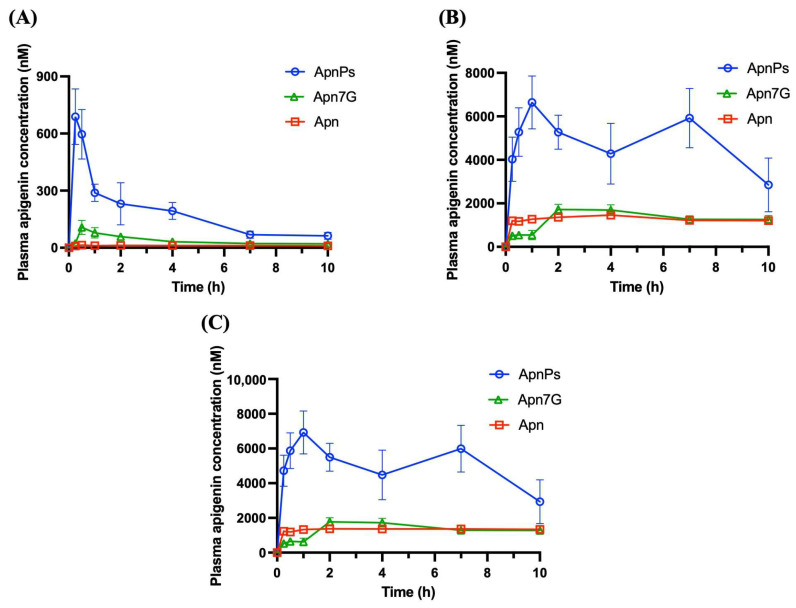
Mean plasma concentration–time profiles of (**A**) aglyconic Apn, (**B**) Apn conjugates, and (**C**) sum of Apn and its conjugates in rats after the oral administration of Apn, Apn7G suspension, and ApnPs solution at 185.02 μmol/kg B.W. Data are the mean ± SE (*n* = 3–4). Apn, apigenin; ApnPs, apigenin phosphate derivatives; and Apn7G, apigenin 7-*O*-glucoside.

Figure 5A demonstrates that the ApnPs group, similarly to LutPs group, had the highest plasma levels, displaying approximately 15.4- and 4.7-fold higher AUC_0–t_ and 49.1- and 6.4-fold higher C_max_ values compared to the Apn and Apn7G group, respectively (Table 3). Moreover, ApnPs displayed rapid absorption with the shortest T_max_ value of 0.25 h, in contrast to the delayed absorption seen in the Apn7G and Apn groups, both of which exhibited a T_max_ of 0.5 h. Figure 5B presents the plasma levels of phase II metabolites of Apn for each group, following similar trends to those of aglyconic Apn. These observations indicate a parallel metabolic behavior to Lut, with most Apn undergoing transformation by phase II enzymes into conjugates.

**Figure 6:**
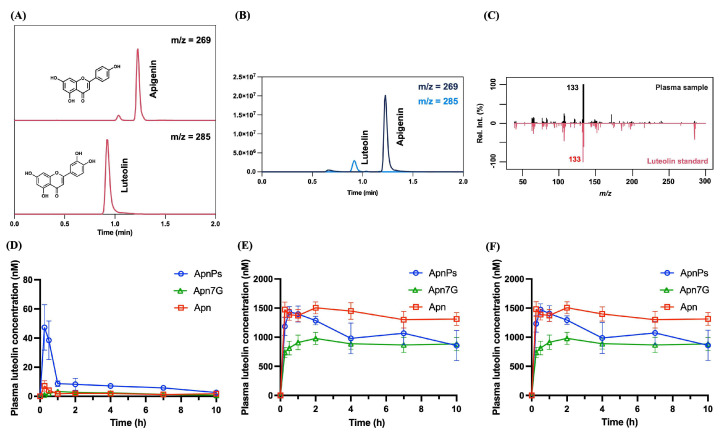
MRM chromatograms of (**A**) standards (200 ng/mL) for Apn (**top**) and Lut (**bottom**) and (**B**) overlay of MRM chromatograms for Apn (*m*/*z* 269) and its metabolite Lut (*m*/*z* 285) in plasma. (**C**) UPLC-MS/MS spectrum of the Lut qualifier ion (*m*/*z* 133) from rat plasma. Mean plasma concentration–time profiles of (**D**) aglyconic Lut, (**E**) Lut conjugates, and (**F**) total Lut (Lut and its conjugates) in rats following the oral administration of Apn, Apn7G suspension, and ApnPs solution at 185.02 μmol/kg B.W. Data are the mean ± SE (*n* = 3−4). Lut, luteolin; Apn, apigenin; ApnPs, apigenin phosphate derivatives; and Apn7G, apigenin 7-*O*-glucoside.

Surprisingly, one notable discovery in this study was the detection of Lut in the plasma after the administration of Apn and its derivatives. This unexpected result was verified using LC-MS/MS (Figure 6A–C). The metabolite Lut exhibited a similar pattern to native Apn in rat plasma, as shown in Figure 6D–F, and was also conjugated by phase II metabolism. Notably, the conversion of Apn to luteolin in the Apn group was approximately 14.3%, as determined by comparing the AUC_0–t_ values of the luteolin metabolite to those of Apn.

## 4. Discussion

Lut and Apn have garnered widespread attention for their diverse bioactivities, including enhanced antioxidant defense [4,8], neuroprotective effects [36,37], and anticancer activity [38,39,40]. However, previous studies have indicated that they exhibit extremely low solubility in aqueous media [41,42], resulting in poor oral bioavailability. In this study, LutPs and ApnPs significantly improved water solubility and dissolution rates, enabling them to dissolve rapidly in bodily fluids and be efficiently hydrolyzed by ALP to release their parent forms, ready to be absorbed. These findings suggest that phosphorylated flavones can effectively overcome the previous limitations.

The pharmacokinetic profiles observed in this study reveal that these flavone monophosphates not only enhance the overall bioavailability of their parent compounds but also exhibit rapid absorption, characterized by shorter T_max_ and significantly higher C_max_ following oral administration (Table 2 and Table 3). This trend aligns with their respective dissolution profiles. In addition to the aglyconic forms, we evaluated the systemic levels of the first-pass metabolites of Lut and Apn. As shown in Figure 4 and Figure 5, the content of the glucuronide/sulfate conjugates of Lut and Apn is significantly higher than that of the aglycones, indicating rapid first-pass metabolism with extensive sulfation and glucuronidation during absorption. These findings are consistent with previous investigations [43,44,45,46], suggesting that glucuronide/sulfate conjugates are the major circulating forms of Lut and Apn in vivo. Previous in vitro studies have shown that Apn undergoes phase II metabolism to form three types of glucuronides and one sulfate product [45,46]. In humans, after oral administration, the primary metabolites identified are apigenin 7-*O*-glucuronide, apigenin 4′-*O*-glucuronide, and apigenin 4′-*O*-sulfate [13]. These phase II metabolites are reported to possess distinct bioactivities. Research indicates that Lut glucuronides, particularly luteolin 7-*O*-glucuronide and luteolin 3′-*O*-glucuronide, significantly reduce the expression of inflammatory genes, with effects comparable to those of Lut itself [43,47]. Similarly, apigenin 7-*O*-glucuronide has been shown to attenuate inflammatory responses by downregulating inflammation-related gene expression and exhibiting antiviral properties. Consequently, these metabolites are considered to contribute to the health benefits associated with the oral administration of Lut and Apn [48,49].

This study is the first to demonstrate the in vivo biotransformation of Apn to Lut via cytochrome P450 monooxygenases (CYP450). Previous studies included two related investigations: an in vitro liver microsome incubation study and an in vivo oral administration study of Apn [45,46]. In the in vitro study, Apn underwent phase I metabolism mediated by CYP1A1, CYP2B, CYP2E1, and NADPH, producing three mono-hydroxylated derivatives. Notably, Lut emerged as the predominant metabolite after 15 min of incubation. Phase II metabolism also produced sulfates, monoglucuronides, and methyl conjugates of both Apn and Lut, indicating a significant in vitro biotransformation from Apn to Lut. However, the subsequent in vivo study found no trace of Lut or related metabolites in plasma, urine, or fecal samples. In contrast, our current study provides the most direct in vivo evidence to date that Apn is transformed into Lut, which is detectable in systemic circulation (Figure 6). The phase I metabolite of Apn was characterized using UPLC-MS/MS spectrum analysis and its MS/MS spectrum was compared to the Lut standard. This finding clarifies longstanding uncertainties about the metabolic relationship between Apn and Lut, advancing our understanding of their metabolic pathways.

## 5. Conclusions

In this study, we used the bacterial strain *Bacillus subtilis* natto BCRC 80517 to produce LutPs and ApnPs. These microbial phosphorylation products not only significantly enhanced water solubility—enabling rapid absorption and increased plasma concentrations of Lut, Apn, and their phase II metabolites—but also provided insight into the biological fate of these flavones. Consequently, LutPs and ApnPs may hold promise as alternatives to Lut and Apn, with potential applications in dietary supplements and pharmacology. Further studies are necessary to confirm their biological activity and evaluate their therapeutic potential.

## Figures and Tables

**Table 1:** Aqueous solubility and cLogP values of the tested substances.

Tested Substances ^a^	Aqueous Solubility ^b^ (μg/mL)	cLogP ^c^
LutPs	1.8 × 10^4^	
Lut7P		0.44
Lut3′P		0.37
Lut4′P		0.37
Lut	1.3 × 10^1^	1.78
Lut7G	5.4 × 10^1^	0.28
ApnPs	1.8 × 10^4^	
Apn7P		1.04
Apn4′P		1.04
Apn	1.2 × 10^0^	2.38
Apn7G	6.9 × 10^1^	0.88

Note: ^a^ Lut, luteolin; LutPs, luteolin phosphate derivatives; Lut7G, luteolin 7-*O*-glucoside; Apn, apigenin; ApnPs, apigenin phosphate derivatives; and Apn7G, apigenin 7-*O*-glucoside. ^b^ The solubility was tested by dissolving samples in ultrapure water (≥18 MΩ·cm) at 25 °C. ^c^ clogP values obtained from ChemDraw Professional 16.0 using the compound’s structure.

**Table 2:** Pharmacokinetic parameters of plasma luteolin, luteolin conjugates, and overall luteolin with the oral administration of tested substances to rats.

	Parameters	Lut	Lut7G	LutPs
aglyconic luteolin	T_max_ (h)	1.00	1.00	0.25
	C_max_ (nM)	139.91 ± 193.96	749.71 ± 571.00	3005.33 ± 170.99
	AUC_0–t_ (nM·h)	635.00	2009.35	5441.02
conjugates	T_max_ (h)	8.00	4.00	4.00
	C_max_ (nM)	4178.78 ± 695.78	4904.73 ± 1703.25	11,865.30 ± 1567.45
	AUC_0–t_ (nM·h)	26,762.14	31,336.03	70,161.75
overall	T_max_ (h)	1.00	2.00	1.00
	C_max_ (nM)	3947.45 ± 740.01	5203.57 ± 384.04	13,923.22 ± 2007.41
	AUC_0–t_ (nM·h)	28,347.76	33,111.44	80,371.46

Note: Data are represented as the mean ± SE (*n* = 3). C_max_, maximum plasma concentration; T_max_, time to maximum plasma concentration; AUC_0–t_, area under the concentration–time curve from time zero to 8 h; Lut, luteolin; LutPs, luteolin phosphate derivatives; and Lut7G, luteolin 7-*O*-glucoside.

**Table 3:** Pharmacokinetic parameters of apigenin, apigenin conjugates, overall apigenin, luteolin (metabolite), luteolin conjugates, and overall luteolin with oral administration of tested substances to rats.

	Parameters	Apn		Apn7G		ApnPs	
		Apn	Lut (Metabolite)	Apn	Lut (Metabolite)	Apn	Lut (Metabolite)
aglyconic form	T_max_ (h)	0.5	0.25	0.5	1.00	0.25	0.25
	C_max_ (nM)	14.02 ± 1.27	7.14 ± 3.65	107.38 ± 37.37	3.22 ± 1.52	688.44 ± 145.45	43.30 ± 15.57
	AUC_0–t_ (nM·h)	112.91	18.8	371.71	18.54	1743.87	84.09
conjugates	T_max_ (h)	4.00	2.00	2.00	2.00	1.00	0.5
	C_max_ (nM)	1456.25 ± 127.23	1504.61 ± 101.91	1718.13 ± 235.57	978.66 ± 104.92	6638.24 ± 1217.89	1436.15 ± 89.57
	AUC_0–t_ (nM·h)	12,833.31	13,672.08	13,243.27	8788.63	48,599.48	10,758.80
overall	T_max_ (h)	2.00	2.00	2.00	2.00	1.00	0.5
	C_max_ (nM)	1371.03 ± 76.08	1506.66 ± 102.40	1776.48 ± 237.17	981 ± 104.32	6927.39 ± 1236.64	1474.62 ± 99.55
	AUC_0–t_ (nM·h)	13,314.82	13,559.48	13,614.98	8870.17	50,385.91	11,301.71

Data are represented as the mean ± SE (*n* = 3–4). C_max_, maximum plasma concentration; T_max_, time to maximum plasma concentration; AUC_0–t_, area under the concentration–time curve from time 0 to 10 h; Apn, apigenin; ApnPs, apigenin phosphate derivatives; and Apn7G, apigenin 7-*O*-glucoside.

## Data Availability

All data are contained within this article.

## Supplementary Materials

The following supporting information can be downloaded at https://www.mdpi.com/article/10.3390/antiox13121530/s1: Table S1: The elution gradient programs of HPLC used in this study; Table S2: MRM conditions for the determination apigenin, luteolin, and genistein (internal standard) by UPLC-MS/MS; Table S3: The validation parameters and results of the UPLC-MS/MS method used in this study; and Table S4: Calibration curve parameters for standard compounds in the HPLC method used in this study.
