# Supplementary material for: Monophosphate Derivatives of Luteolin and Apigenin as Efficient Precursors with Improved Oral Bioavailability in Rats
Source: Antioxidants (Basel). 2024 Dec 13;13(12):1530. doi: 10.3390/antiox13121530 (PMC11727040; doi:10.3390/antiox13121530)
Supplement: Supplementary file 1 [file antioxidants-13-01530-s001.zip › antioxidants-3288536-supplementary.pdf]

Supplementary material

Monophosphate Derivatives of Luteolin and Apigenin as Efficient Precursors with Improved Oral Bioavailability in Rats

Sydney Wu[1,†], Shang-Ta Wang[2,3,†], Guan-Yuan Chen[4,5,†], Chen Hsu[1], Yi-Hsin Chen[6], Hsin-Ya Tsai[1], Te-I Weng[4,5,7], Chien-Li Chen[2], Yi-Fang Wu[4,8], and Nan-Wei Su[1,6*]

[1] Department of Agricultural Chemistry, National Taiwan University, Taipei 106, Taiwan
[2] Department of Food Science, National Taiwan Ocean University, Keelung 202, Taiwan
[3] Institute of Food Safety and Risk Management, National Taiwan Ocean University, Keelung 202, Taiwan
[4] Forensic and Clinical Toxicology Center, National Taiwan University Hospital, Taipei 100, Taiwan
[5] Department and Graduate Institute of Forensic Medicine, College of Medicine, National Taiwan University, Taipei 100, Taiwan
[6] Department of Biochemical Science & Technology, National Taiwan University, Taipei 106, Taiwan
[7] Department of Emergency Medicine, National Taiwan University Hospital, 100, Taiwan
[8] School of Pharmacy, College of Medicine, National Taiwan University, Taipei, Taiwan

* Correspondence: snw@ntu.edu.tw
† These authors contributed equally to this work.

Supplementary Tables

Table S1. The elution gradient programs of HPLC used in this study

	Time (min)	Solvent A (%) 0.1 % phosphoric acid in H_2O	Solvent B (%) 0.1 % phosphoric acid in acetonitrile
Luteolin, LutPs, and Lut7G			
	0	85	15
	5	75	25
	10	60	40
	20	55	45
	25	85	15
	30	85	15
Apigenin, ApnPs, and Apn7G			
	0	85	15
	25	60	40
	30	10	90
	35	85	15
	45	85	15
Fosphenytoin			
	0-20	50	50 (isocratic)

Flow rate was set at 1 mL/min. LutPs, luteolin phosphate derivatives; Lut7G, luteolin 7-*O*-glucoside; ApnPs, apigenin phosphate derivatives; Apn7G, apigenin 7-*O*-glucoside.

Table S2. MRM conditions for determination apigenin, luteolin, and genistein (internal standard) by UPLC-MS/MS

Name	Precursor ion (m/z)	Product ion (m/z)	Cone voltage (V)	Collision energy (V)	Retention time (min)
Apigenin	269	65<br>107<br>117	4	44<br>30<br>38	1.23
Luteolin	285	65<br>133<br>151	54	38<br>34<br>22	0.93
Genistein (IS	269	41<br>63<br>133	50	50<br>34<br>24	1

Table S3. The validation parameters and results of UPLC-MS/MS method used in this study

Analytes	Linearity	R^2	Concentration range (ng/mL)	
Luteolin	y = 0.0180x+0.00241	0.998	0.5-1000	
Apigenin	y = 0.0201x-0.00639	0.999	0.5-1000	
		Concentration (ng/mL)	Precision (RSD%)	Accuracy (%)
Luteolin	LQC	4.63	2.69	93
	MQC	47.0	2.16	94
	HQC	434.1	2.39	87
Apigenin	LQC	5.03	4.68	101
	MQC	51.1	4.20	102
	HQC	510.2	1.16	102

LQC, low-quality control; MQC, medium-quality control; HQC, high-quality control; RSD, relative standard deviation

Table S4. Calibration curve parameters for standard compounds in the HPLC method used in this study

Analytes	Concentration range (μM)	Linearity	R^2
Lut	0.25-20	y = 28094x+2938.3	0.99
Lut7P	0.8-7.00	y = 13595x+2985	0.99
Lut3′P	0.5-45	y = 15145x+875.61	0.99
Lut4′P	0.08-7.35	y = 15141x+139.68	0.99
Lut7G	0.25-55	y = 16835x+3751.9	0.99
Apn	0.25-50	y = 23539x+3087.8	0.99
Apn7P	0.25-50	y = 19044x+1566.9	0.99
Apn4′P	0.25-50	y = 24395x+5016.1	0.99
Apn7G	0.125-50	y = 60165x+6877.9	0.99
Fosphenytoin	0.5-55	y = 14406x+3660.3	0.99

Lut, luteolin; Lut7P, luteolin 7-*O*-phosphate; Lut3′P, luteolin 3′-*O*-phosphate; Lut4′P, luteolin 4′-*O*-phosphate; Lut7G, luteolin 7-*O*-glucoside. Apn, apigenin; Apn4′P, apigenin 4′-*O*-phosphate; Apn7′P, apigenin 7′-*O*-phosphate; Apn7G, apigenin 7-*O*-glucoside
